# Comparing waist circumference with body mass index on obesity-related cancer risk: a pooled Swedish study

**DOI:** 10.1093/jnci/djaf075

**Published:** 2025-03-28

**Authors:** Ming Sun, Christel Häggström, Marisa da Silva, Innocent B Mboya, Ylva Trolle Lagerros, Karl Michaëlsson, Sven Sandin, Jerzy Leppert, Sara Hägg, Sölve Elmståhl, Patrik K E Magnusson, Stefan Söderberg, Weiyao Yin, Abbas Chabok, Angela Wood, Tanja Stocks, Josef Fritz

**Affiliations:** Department of Translational Medicine, Lund University, Malmö, Sweden; Northern Registry Centre, Department of Diagnostics and Intervention, Oncology, Umeå University, Umeå, Sweden; Department of Translational Medicine, Lund University, Malmö, Sweden; School of Information Technology, Halmstad University, Halmstad, Sweden; Department of Translational Medicine, Lund University, Malmö, Sweden; Africa Academy for Public Health (AAPH), Dar es Salaam, Tanzania; Department of Epidemiology and Biostatistics, Institute of Public Health, Kilimanjaro Christian Medical University College, Moshi, Tanzania; Department of Medicine, Huddinge, Karolinska Institutet, Stockholm, Sweden; Medical Epidemiology, Department of Surgical Sciences, Uppsala University, Uppsala, Sweden; Department of Medical Epidemiology and Biostatistics, Karolinska Institutet, Stockholm, Sweden; Department of Psychiatry, Icahn School of Medicine at Mount Sinai, New York, NY, United States; Centre for Clinical Research, Västmanland Hospital, Uppsala University, Uppsala, Sweden; Department of Medical Epidemiology and Biostatistics, Karolinska Institutet, Stockholm, Sweden; Department of Clinical Sciences Malmö, Lund University, Malmö, Sweden; Department of Medical Epidemiology and Biostatistics, Karolinska Institutet, Stockholm, Sweden; Department of Public Health and Clinical Medicine, Umeå University, Umeå, Sweden; Department of Medical Epidemiology and Biostatistics, Karolinska Institutet, Stockholm, Sweden; Department of Clinical Sciences, Division of Surgery, Danderyd Hospital, Karolinska Institutet, Stockholm, Sweden; Department of Public Health and Primary Care, University of Cambridge, Cambridge, United Kingdom; Department of Translational Medicine, Lund University, Malmö, Sweden; Department of Translational Medicine, Lund University, Malmö, Sweden; Institute of Clinical Epidemiology, Public Health, Health Economics, Medical Statistics and Informatics, Medical University of Innsbruck, Innsbruck, Austria

## Abstract

**Background:**

General adiposity, assessed by body mass index (BMI), is a well-established cancer risk factor. This study compared waist circumference (WC), a measure of abdominal adiposity, with BMI as a risk factor for obesity-related cancers, and assessed whether WC provides additional information beyond BMI.

**Methods:**

We analyzed data from 339 190 individuals in a pooled Swedish cohort with baseline BMI and WC assessments from 1981 to 2019 (61% objectively measured, mean age 51.4 years). Cancer diagnoses were obtained from the Swedish Cancer Register. Hazard ratios (HRs) for WC and BMI were calculated using multivariable-adjusted Cox regression. To account for WC’s greater variability, we corrected HRs using regression dilution ratios. To assess WC’s additional contribution beyond BMI, we analyzed WC residuals in multivariable, BMI-adjusted models.

**Results:**

During a median follow-up of 13.9 years (interquartile range: 8.0-22.5), 18 185 IARC-established obesity-related cancers were recorded. In men, a 1-standard deviation (SD) increase in WC was associated with a 25% higher risk of obesity-related cancers (HR_1-SD_ = 1.25, 95% CI = 1.21 to 1.30), compared to a 19% increase for BMI (HR_1-SD_ = 1.19, 95% CI = 1.15 to 1.23, *P* = 0.014 for heterogeneity). Among women, associations were weaker and similar for both WC (HR_1-SD_ = 1.13, 95% CI = 1.11 to 1.16) and BMI (HR_1-SD_ = 1.13, 95% CI = 1.11 to 1.15, *P* = 0.357 for heterogeneity). Waist circumference residuals were more strongly associated with obesity-related cancer risk in men (HR_1-SD_ = 1.09, 95% CI = 1.06 to 1.12) than in women (HR_1-SD_ = 1.03, 95% CI = 1.02 to 1.05). Including an additional 6893 potential obesity-related cancers yielded similar patterns of associations.

**Conclusion(s):**

Waist circumference is a stronger risk factor than BMI for obesity-related cancer in men, conveying additional risk information, whereas this is less evident in women.

## Introduction

Convincing evidence links adiposity, as measured by body mass index (BMI), to at least 13 different cancer sites, with emerging research suggesting that even more cancer sites and some morphological subtypes, including some rarer ones, may be affected.^1-3^ However, BMI, calculated as weight in kilograms divided by the square of height in meters, does not differentiate between fat mass and lean mass, nor does it account for the distribution of body fat. It has been suggested that central adiposity might be a more relevant risk factor for certain cancers than overall adiposity as measured by BMI.^4^^,^^5^

Waist circumference (WC) is a simple and cost-effective anthropometric measure that reflects abdominal fat accumulation, including both intraperitoneal (visceral) and subcutaneous fat. It is frequently used as a proxy for central adiposity.^6^^,^^7^ Body mass index is a measure of body size and although strongly correlated with WC (ρ_Pearson_∼0.9),^8^ WC more specifically measures abdominal fat mass, which is metabolically active and has been linked to cancer development and progression through mechanisms such as insulin resistance, chronic inflammation, and hormonal dysregulation.^9-11^ Therefore, WC may provide additional information related to cancer risk beyond what BMI offers.

However, in contrast to BMI, the effects of WC on specific cancers remain less researched. Only a limited number of large studies address WC and the risk of specific forms of cancer to date.^5^^,^^12-14^ In this study, we aimed to compare the relationships between WC and the risk of developing different cancers, all of which have an established or potential association with obesity, with those of BMI, separately for men and women (aim 1). Additionally, we sought to determine whether WC provides additional risk information beyond that already captured by BMI (aim 2).

## Methods

### Study population

We used data from the Obesity and Disease Development Sweden (ODDS) study, which pools large Swedish cohorts and national registers.^15^ This dataset includes individual-level information on height, weight, and additionally WC in a subpopulation (objectively measured or self-reported once or more). Information on smoking habits was collected in some of the cohorts. The study was performed in line with the principles of the Declaration of Helsinki and was approved by the Swedish Ethical Review Authority (no: 2020-03846).

### Register linkages

Individuals in the study population were linked with data from several national registries by record linkages using the unique personal identity number assigned to each resident of Sweden.^16^ We retrieved information on date of death from the Cause of Death Register,^17^ sex, date of birth and emigration, country of birth, and marital status from the Total Population Register. Furthermore, we retrieved education level, total income per year, and main source of income from the longitudinal integrated database for health insurance and labor market studies (LISA).^15^

Cancer diagnoses were identified by linking to the Swedish Cancer Register. The register is based on mandatory reporting of cancer diagnoses of the population since 1958 and is estimated to capture more than 95% of cancer diagnoses in Sweden.^18^

We classified cancers using International Classification of Diseases (ICD) codes, WHO/HS/CANC/24.1 (Swedish PAD codes), and ICD-O-2 and -O-3 codes (Swedish SNOMED codes), as previously described.^3^ First-incident primary malignancies of established or potential obesity-related cancers were included as outcomes.

Established obesity-related cancers were defined as those for which the International Agency for Research on Cancer (IARC) has concluded there is sufficient evidence linking them to obesity, including cancers of the esophagus (adenocarcinoma), gastric (cardia), colon, rectum, liver/intrahepatic bile ducts, gallbladder, pancreas, breast (postmenopausal), endometrium, ovary, renal cell carcinoma, meningioma, thyroid, and multiple myeloma (14 different cancers in our study; colon and rectum cancer were originally not separated).^1^

Potential obesity-related cancers were defined as those positively associated with obesity (BMI ≥30 kg/m^2^) vs normal weight (BMI 18.5-24.9 kg/m^2^), or with continuous BMI, in our large Swedish study that included the present study population.^3^ Those potential obesity-related cancers made up 15% of incident cancers in our recent investigation,^3^ and included cancers of the oral cavity, nasal and paranasal sinuses, gastric (gastrointestinal stromal tumours), small intestine, biliary tract, pancreatic islets, adrenal glands, parathyroid gland, pituitary gland, connective tissue, lymphoid neoplasms, and myeloid neoplasms (all for both men and women), cancers of the head and neck (adenocarcinoma), penis, and malignant melanoma (only for men), and cancers of the head and neck (squamous-cell carcinoma), nodular melanoma, vulva, and cervix (adenocarcinoma) (only for women). Detailed definitions are provided in Table S1.

### Participant selection

We selected individuals for the study from a total of 363 934 individuals in ODDS with 511 453 assessments of WC, weight, and height. We excluded assessments with extreme values of WC (<40 or >160 cm), weight (<35 or >250 kg), height (<100 or >250 cm), or BMI (<15 or >60 kg/m^2^; in total 333 individuals/820 assessments), mismatched dates (11 241 individuals/15 558 assessments), or a prior cancer diagnosis (13 170 individuals/26 653 assessments). This resulted in a study population of 339 190 individuals, with the first assessment defined as baseline (Figure S1).

### Statistical analysis

To assess the risk associated with higher WC and BMI (as measured at baseline) in relation to obesity-related cancers, we calculated hazard ratios (HRs) with 95% confidence intervals (CIs) from multivariable-adjusted Cox regression models with attained age as the underlying time metric. We counted person-years at risk from baseline (or from age 55 years for the analysis of postmenopausal breast cancer) until the diagnosis of an obesity-related cancer, censoring at the date of another prior cancer, death, emigration, or end of follow-up on December 31, 2019, whichever came first. For a few cancers that could only be correctly classified using ICD10 or ICD-O-2 codes, rather than ICD7 (Table S1), we started follow-up on January 1, 1993, when these later ICD versions were introduced, or at baseline, whichever came later.

Models were fit to men and women combined, as well as separately for men and women, stratified by calendar year of birth (<1940, 1940-1949, 1950-1959, 1960-1969, ≥1970) and sex (in the combined analysis), and adjusted for baseline age (continuous), smoking status, cohort, marital status, education level, birth country, income level, and main source of income. We additionally adjusted for the mode of WC assessment (objectively measured/self-reported) and height (continuous) for the analysis of WC, and mode of weight and height assessment (objectively measured/self-reported) for the analysis of BMI. Missing data for smoking status, education level, income level, and main source of income were treated as separate categories. In all analyses, potential sex interactions were examined using Wald tests by including product terms of sex and WC or BMI into the respective model.

Individual cancer sites were only analyzed if the number of cases was at least 100. We used Schoenfeld residual statistics to check the proportional hazards assumption of the Cox models. While we detected slight deviations for smoking status, incorporating it as a strata did not change the obtained HRs. Therefore, we chose to simply adjust for smoking status in our models.

We used restricted cubic splines Cox models to visualize the shape of association of WC with cancer risk across the whole range of WC values. The linearity of the splines was evaluated by testing if the set of spline coefficients beyond the linear term was collectively zero with a postestimation Wald test.

For aim 1, to facilitate a comparison of the strength of association between WC and BMI with cancer risk, we analyzed HRs per 1-standard deviation (SD) increase of WC and BMI, with WC standardized within sex strata. Given that HRs might be diluted by intrapersonal variability and random measurement error, we corrected the continuous HRs of WC and BMI using a method based on regression dilution ratios (RDRs) as described by Wood et al., utilizing all available repeated WC and/or BMI measurements.^19^ Repeated measurements were available for 37 754 men and 68 073 women. Details about the obtained RDRs are shown in Figure S2. All HRs as originally obtained from the Cox models were corrected via the formula ${\mathrm{HR}}_{\mathrm{corrected}}=\exp(\log\left({\mathrm{HR}}_{\mathrm{original}}\right)/\mathrm{RDR})$. For obesity-related cancers combined, we also analyzed HRs for WC and BMI in sex-specific quintiles. The χ^2^ statistic for trend across quintile medians was used to quantify the strength of the relationship between WC and BMI and cancer risk. Heterogeneity between the HRs for WC and BMI was tested using the Wald test, with the test statistic defined as $\left(\beta_{WC}-\beta_{\mathrm{BMI}}\right)/\left(\mathrm{Var}\left(\beta_{WC}\right)+\mathrm{Var}\left(\beta_{\mathrm{BMI}}\right)-2\;\times \;\mathrm{Cov}(\beta_{WC},\beta_{\mathrm{BMI}})\right)$, where β represents the log-transformed HRs. The covariance term, $\mathrm{Cov}(\beta_{WC},\beta_{\mathrm{BMI}})$, was estimated using seemingly unrelated estimation (SUEST).^20^ Sensitivity analyses restricted to never-smokers and to smoking-related cancer sites (as defined by Sun et al.^3^) were conducted to evaluate the robustness of our main findings. Additionally, subgroup analyses were also conducted separately for participants with objectively measured and self-reported WC and BMI.

For aim 2, we explored the additional contribution of WC as a cancer risk factor beyond BMI using 3 approaches. First, we performed residual analyses for BMI and WC, where WC was regressed on BMI in sex-specific, multivariable-adjusted linear regression models. The residuals from these models were then included in a multivariable, BMI-adjusted Cox model.^12^^,^^21^ The HRs of the residuals quantify whether, and to what extent, WC provides additional risk information beyond BMI.

Second, we created tertiles of BMI and WC within each BMI tertile of the population. Calculation of HRs of BMI tertiles within its population tertiles estimated the residual effect of the variation of BMI within BMI tertiles. If the HRs for WC tertiles within BMI population tertiles are larger than that of BMI tertiles within BMI population tertiles, this suggests that WC provides additional risk information beyond BMI. Conversely, if the effect sizes are comparable, it indicates that WC does not contribute extra risk information.

Third, we calculated HRs for continuous WC within BMI quintiles, and HRs for continuous BMI within WC quintiles, per 1-SD increase. If the HRs for WC from the first model are similar in magnitude to the HRs for BMI from the second model, it indicates that BMI and WC contribute similarly as cancer risk factors beyond the influence of the other. Conversely, if WC contributes more to BMI than vice versa, the HRs for WC from the first model should be higher than the HRs for BMI from the second model. We chose not to mutually adjust WC and BMI due to their high correlation (ρ_Pearson_ = 0.83 in men and 0.81 in women).

All analyses were performed using Stata 18.0 (StataCorp LLC, College Station, TX), with all tests being 2-sided and a significance level set at 0.05.

## Results

### Study population

A total of 339 190 individuals, 142 434 men and 196 756 women, with a mean baseline age of 53.1 (SD 12.4) and 50.2 (SD 13.1) years, were eligible for analysis. Mean (SD) values of WC were 95.5 (10.8) cm in men and 82.3 (11.8) cm in women; for BMI, mean (SD) values were 26.3 (3.7) kg/m^2^ in men, and 24.9 (4.3) kg/m^2^ in women (Table 1). After a median follow-up of 13.9 years (interquartile range [IQR] 8.0-22.5), 18 185 established obesity-related cancers (4482 in men and 13 703 in women [thereof 6851 postmenopausal female breast cancers]) and 6893 potential obesity-related cancers (3802 in men and 3091 in women) had been recorded.

**Table 1.** Baseline characteristics of the study population.

Characteristics	Men (*n* = 142 434)	Women (*n* = 196 756)	Total (*n* = 339 190)
**Cohort (health examinations, range of years), *n* (%)**			
Västerbotten Intervention Programme (2003-2019)	39 868 (28)	39 069 (20)	78 937 (23)
Women’s Lifestyle and Health (1991, 1992, 2003)	0 (0)	40 287 (21)	40 287 (12)
Cohort of Swedish Men (1997, 2008, 2019)	35 059 (25)	0 (0)	35 059 (10)
Swedish Mammography Cohort (1997, 2008, 2019)	0 (0)	30 998 (16)	30 998 (9)
Westmannia Cardiovascular Risk Factors Study (1989-2000)	15 034 (11)	14 311 (7)	29 345 (9)
Swedish National March Cohort (1997)	9072 (6)	18 254 (9)	27 326 (8)
LifeGene (2009-2016)	10 504 (7)	15 210 (7)	25 714 (8)
Malmö Diet and Cancer Study (1991-1996)	9370 (7)	15 617 (8)	24 987 (8)
EpiHealth (2011-2018)	8722 (6)	8656 (4)	17 378 (5)
Northern Sweden Monica Project (1986-2014)	5497 (4)	5515 (3)	11 012 (3)
TwinGene (2002-2009)	4639 (3)	5469 (3)	10 108 (3)
Malmö Preventive Project (1981-1992)	2853 (2)	1453 (1)	4306 (1)
Malmö Offspring Study (2013-2019)	1816 (1)	1917 (1)	3733 (1)
**Year of health examination, median (range)**	1999 (1981-2019)	1997 (1986-2019)	1997 (1981-2019)
**Age, years**			
Mean (SD)	53.1 (12.4)	50.2 (13.1)	51.4 (12.9)
Category, *n* (%)			
<30	5 470 (4)	10 072 (5)	15 542 (4)
30-44	32 031 (22)	61 756 (31)	93 787 (28)
45-59	61 103 (43)	76 917 (39)	138 020 (41)
≥60	43 830 (31)	48 011 (25)	91 841 (27)
**Waist circumference, cm**			
Mean (SD)	95.5 (10.8)	82.3 (11.8)	87.8 (13.1)
Category, *n* (%)			
<94 in men, <80 in women	64 281 (45)	91 407 (46)	155 688 (46)
94-102 in men, 80-88 in women	46 185 (32)	54 705 (28)	100 890 (30)
102 in men, >88 in women	31 968 (23)	50 644 (26)	82 612 (24)
**Waist circumference measurement, *n* (%)**			
Objectively measured	98 303 (69)	107 217 (54)	205 520 (61)
Self-reported	44 131 (31)	89 539 (46)	133 670 (39)
**Height, mean (SD), cm**	178.3 (6.8)	165.2 (6.1)	170.7 (9.1)
**Weight, mean (SD), kg**	83.5 (13.1)	68.0 (12.2)	74.5 (14.7)
**Body mass index, kg/m** ^2^			
Mean (SD)	26.3 (3.7)	24.9 (4.3)	25.5 (4.1)
Category, *n* (%)			
Underweight (<18.5)	569 (<1)	3 494 (2)	4 063 (1)
Normal weight (18.5-24.9)	56 436 (40)	112 598 (57)	169 034 (50)
Overweight (25.0-29.9)	65 910 (46)	57 210 (29)	123 120 (36)
Obesity (≥30)	19 519 (14)	23 454 (12)	42 973 (13)
**Body mass index measurement, *n* (%)** ^a^			
Objectively measured	98 303 (69)	107 215 (54)	205 518 (61)
Self-reported	44 131 (31)	89 541 (46)	133 672 (39)
**Smoking status, *n* (%)** ^b^			
Never	61 578 (47)	94 224 (53)	155 802 (50)
Former	44 762 (34)	47 481 (27)	92 243 (30)
Current	25 862 (19)	34 683 (20)	60 545 (20)
**Highest achieved education, *n* (%)** ^b^			
Pre-upper secondary school education ≤9 years	33 462 (24)	39 817 (20)	73 279 (22)
Upper secondary school ≤3 years	62 825 (44)	80 465 (41)	143 290 (42)
Post-upper secondary school <3 years	18 532 (13)	27 997 (14)	46 529 (14)
Post-upper secondary school ≥3 years	27 361 (19)	48 224 (25)	75 585 (22)
**Country of origin, *n* (%)**			
Born in Sweden with both parents born in Sweden	123 371 (86)	168 212 (86)	291 583 (86)
Born in Sweden with one or both parents born abroad	6 662 (5)	10 167 (5)	16 829 (5)
Born abroad	12 401 (9)	18 377 (9)	30 778 (9)
**Marital status, *n* (%)**			
Unmarried	34 169 (24)	41 442 (21)	75 611 (22)
Married/registered partnership	89 140 (63)	116 371 (59)	205 511 (61)
Divorced/dissolution of partnership	15 907 (11)	27 035 (14)	42 942 (13)
Widow/-er	3 218 (2)	11 908 (6)	15 126 (4)
**Income level, *n* (%)** ^b^			
Low (<147.0 kSEK/year)	20 493 (16)	49 460 (33)	69 953 (25)
Middle low (147.0-219.0 kSEK/year)	30 358 (23)	39 582 (26)	69 940 (25)
Middle high (219.1-316.8 kSEK/year)	34 748 (27)	35 167 (24)	69 915 (25)
High (≥316.9 kSEK/year)	44 361 (34)	25 571 (17)	69 932 (25)
**Main source of income, *n* (%)** ^b^			
Work	87 557 (67)	98 307 (66)	185 864 (66)
Pension	28 685 (22)	30 280 (20)	58 965 (21)
Other^c^	13 718 (11)	21 193 (14)	34 911 (13)

Abbreviation: SD = standard deviation.

a Weight measurement distribution, which was very similar to that of height.

b Number of individuals with missing values: smoking status, 30 600 (9%); highest achieved education, 507 (<1%); income level, 59 450 (18%); main source of income, 59 450 (18%).

c Other source of income includes studies, care of child/family, sickness, unemployment, early retirement, economic aid, and labor market policy activity.

### WC as cancer risk factor compared to BMI (aim 1)

**Figure 1.**
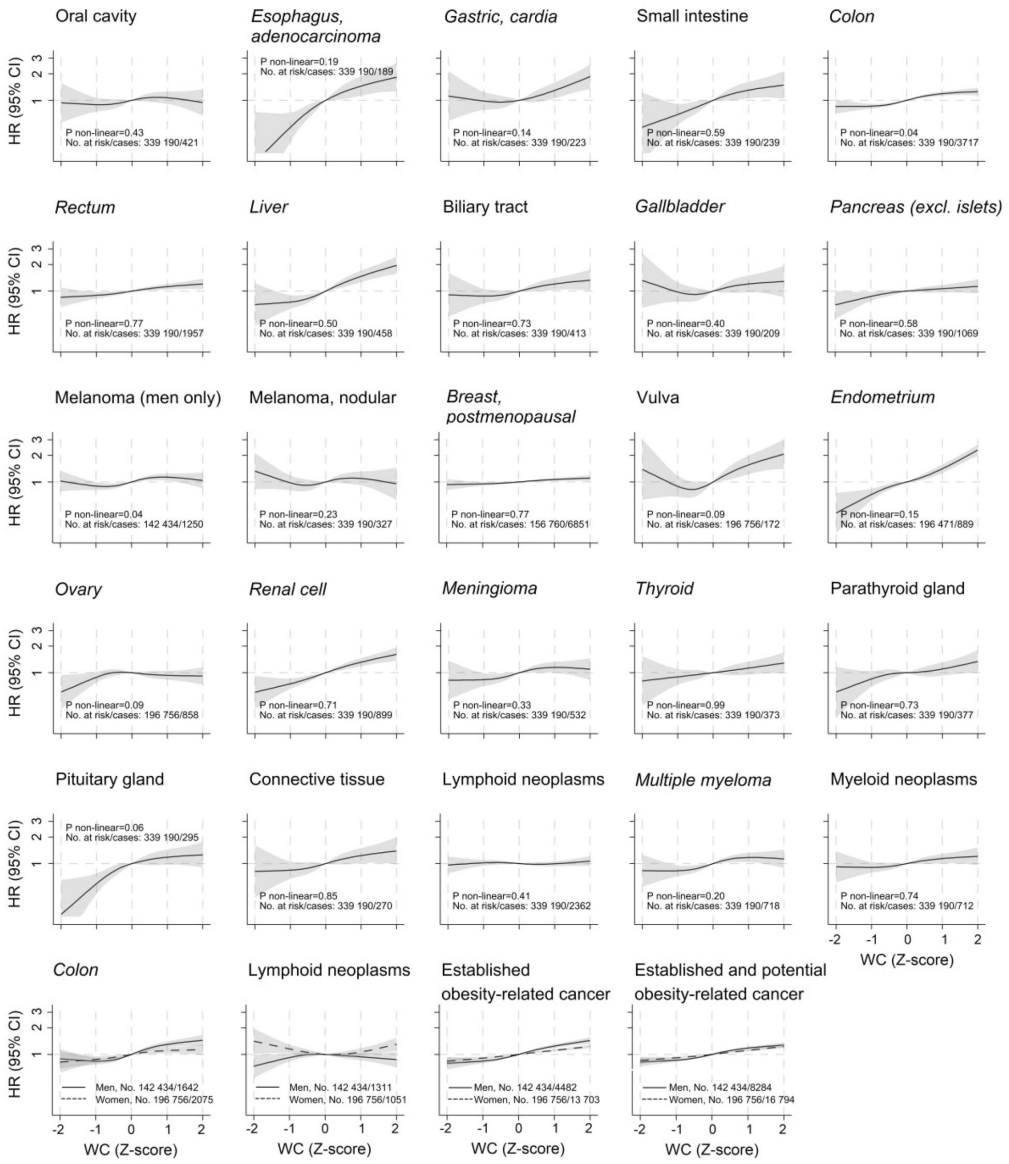
Hazard ratios (95% confidence interval) of each established (in italics) and potential obesity-related cancer according to waist circumference Z-score for men and women combined, and separately in case of a sex interaction (last row), allowing for nonlinear effects, with 95% confidence intervals. Cox models with restricted cubic splines with 4 knots placed at Harrell’s recommended percentiles (ie, 5th, 35th, 65th, and 95th percentile) of waist circumference were fitted stratified by sex and calendar year of birth (<1940, 1940-1949, 1950-1959, 1960-1969, ≥1970), and adjusted for baseline age (continuous), mode of waist circumference assessment, height (continuous), smoking status, cohort, marital status, education level, birth country, income level, and main source of income. Waist circumference as used in the Cox models was standardized within sex strata. The (standardized) reference waist circumference for these plots (with hazard ratio fixed to 1.0) was 0. The absolute waist circumferences values on the *x*-axis at Z-scores −2, −1, 0, 1, and 2 were for men 73.9, 84.7, 95.5, 106.2, and 117.0 cm, and for women 58.7, 70.5, 82.3, 94.0, and 105.8 cm, respectively. Potential obesity-related cancers not presented separately due to a small number of cases (<100) are: nasal and paranasal sinuses, head and neck (adenocarcinoma and squamous-cell carcinoma), gastric (gastrointestinal stromal tumours), cervix (adenocarcinoma), penis, pancreatic islets, and adrenal glands. However, these cancer sites are included in the analysis of all established and potential obesity-related cancers combined.

The estimated shape of the association of WC with cancer risk, allowing for nonlinearity and categorized by cancer sites, is illustrated in Figure 1. Evidence of nonlinearity in those associations was only found for colon cancer and malignant melanoma amongst men. Sex interactions were observed only for colon cancer and lymphoid neoplasms.

**Figure 2.**
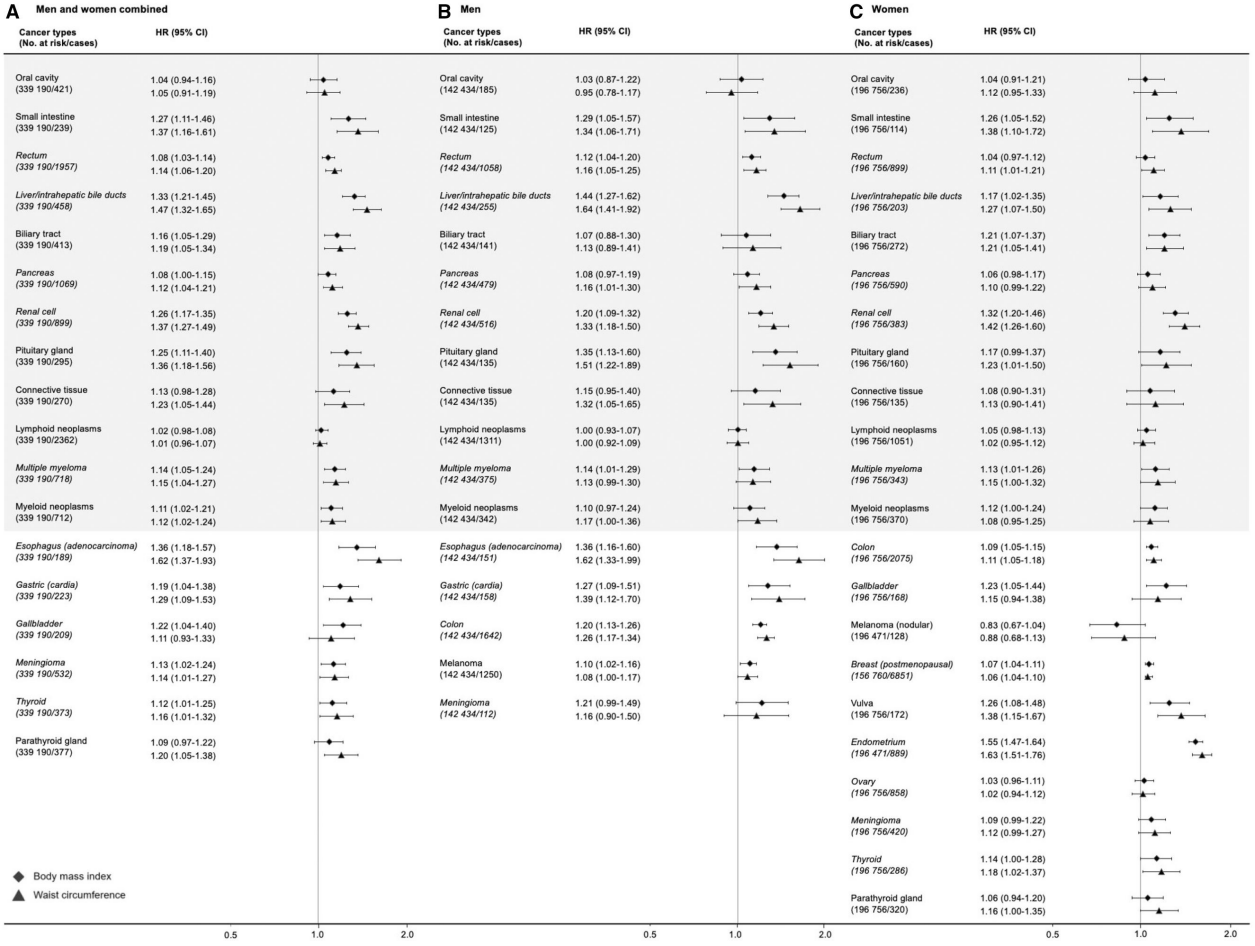
Hazard ratios (95% confidence interval) of each established (in italics) and potential obesity-related cancer per standard deviation higher body mass index and waist circumference, respectively, in men and women combined (**A**), and men (**B**) and women (**C**) separately. Cancers analyzed in both men and women separately are placed at the top and gray shaded for increased comparability. Results for men and women combined are only shown in absence of a sex interaction (all cancers except colon and lymphoid neoplasms). Waist circumference was standardized within sex strata. Hazard ratios were calculated by use of Cox regression using age as time scale, stratified by calendar year of birth (<1940, 1940-1949, 1950-1959, 1960-1969, ≥1970) and sex (in the analysis of men and women combined), and adjusted for baseline age (continuous), smoking status, cohort, marital status, education level, birth country, income level, and main source of income. We additionally adjusted for mode of waist circumference assessment and height (continuous) for the analysis of waist circumference, and mode of weight and height assessment for the analysis of body mass index. Hazard ratios were corrected for regression dilution ratios of body mass index or waist circumference (men and women combined, BMI: 0.93, WC: 0.81; men, BMI: 0.92, WC: 0.78; women, BMI: 0.95, WC: 0.83). Abbreviations: BMI = body mass index; CI = confidence interval; HR = hazard ratio; WC = waist circumference.

When WC and BMI were treated as linear terms in the statistical models, among men a 1-SD increase in WC was associated with a 25% higher risk of developing established obesity-related cancers (HR = 1.25, 95% CI = 1.21 to 1.30). This association was stronger than for BMI, which had an HR of 1.19 (95% CI = 1.15 to 1.23) per 1-SD increase (*P* = 0.014 for heterogeneity, Table 2). Among women, the associations of WC and BMI were weaker and of comparable size (HR per 1-SD increase = 1.13, 95% CI = 1.11 to 1.16 for WC, and 1.13, 95% CI = 1.11 to 1.15 for BMI, *P* = 0.357 for heterogeneity) for established obesity-related cancers. When we excluded postmenopausal breast, endometrial, and ovarian cancer from the analysis of women to ensure comparability of cancer sites between men and women, the results remained largely unchanged (HR per 1-SD increase = 1.13, 95% CI = 1.09 to 1.17 for WC; HR = 1.12, 95% CI = 1.08 to 1.17 for BMI, *P* = 0.093 for heterogeneity). Similar trends of associations to those of established obesity-related cancers were found for all established and potential obesity-related cancers combined, albeit the associations were somewhat weaker in men. Analyses of WC and BMI quintiles in men and women, respectively, confirmed the results of analyses of continuous WC and BMI (Table 2). Sensitivity analyses in never-smokers, for smoking-related cancers, and by mode of measurement (objectively measured vs self-reported data) showed results broadly consistent with the main findings for BMI and WC (Table S2). While HRs for BMI were similar across measurement subgroups, HRs for WC were closer to those for BMI in the self-reported group but remained slightly larger, with a more pronounced difference observed in the objectively measured group. Stratification by cancer site confirmed that BMI and WC were associated with cancer risk for all investigated cancers, except cancers of the oral cavity, lymphoid neoplasms, and ovarian cancer (established obesity-related cancers), and nodular melanoma (a potential obesity-related cancer; Figure 2). Analyses further indicated that HRs for WC were generally slightly higher than those for BMI, with point estimates for HR per 1-SD increase being higher for WC in 12 out of 17 analyzed cancers for men and 16 out of 22 analyzed cancers for women. These differences were most pronounced in cancers strongly associated with obesity (HRs ≥ 1.25 for either WC or BMI), including cancers of the esophagus (adenocarcinoma), gastric (cardia), small intestine, colon (in men), renal cell, liver/intrahepatic bile ducts, pituitary gland, endometrium, and vulva. However, the 95% CIs for HRs per SD always overlapped (Figure 2).

**Table 2.** Hazard ratios (95% confidence interval) of all established obesity-related cancers and all established and potential obesity-related cancers combined according to body mass index and waist circumference level.

Median (Range)		All established obesity-related cancers		All established and potential obesity-related cancers	
		No. at risk/cases	HR (95% CI)^a^	No. at risk/cases	HR (95% CI)^a^
***Men***					
**WC quintiles**					
Q1	83.0 (40.0-86.5)	27 105/554	Ref.	27 105/1101	Ref.
Q2	89.0 (86.7-91.5)	25 642/727	1.16 (1.04 to 1.30)	25 642/1360	1.09 (1.04 to 1.14)
Q3	94.0 (91.6-97.0)	34 383/1118	1.27 (1.14 to 1.40)	34 383/2113	1.17 (1.11 to 1.22)
Q4	100.0 (97.1-103.2)	26 640/965	1.40 (1.26 to 1.56)	26 640/1782	1.19 (1.13 to 1.25)
Q5	109.0 (103.3-160.0)	28 664/1118	1.67 (1.51 to 1.86)	28 664/1928	1.31 (1.25 to 1.38)
***P* for trend/χ^2^**			<.001/220.09		<.001/281.83
**WC per SD increase** ^b^		142 434/4482	1.25 (1.21 to 1.30)^c^	142 434/8284	1.18 (1.16 to 1.22)^c^
**BMI quintiles**					
Q1	22.1 (15.0-23.2)	28 588/766	Ref.	28 588/1479	Ref.
Q2	24.2 (23.3-25.0)	28 391/840	1.05 (0.95 to 1.16)	28 391/1576	1.11 (1.04 to 1.17)
Q3	25.8 (25.1-26.6)	28 516/890	1.11 (1.01 to 1.22)	28 516/1696	1.18 (1.11 to 1.24)
Q4	27.7 (26.7-28.8)	28 453/1000	1.29 (1.17 to 1.41)	28 453/1844	1.23 (1.16 to 1.30)
Q5	30.9 (28.9-60.0)	28 486/986	1.48 (1.35 to 1.63)	28 486/1689	1.35 (1.28 to 1.43)
***P* for trend/χ^2^**			<.001/226.37		<.001/259.23
**BMI per SD increase** ^b^		142 434/4482	1.19 (1.15 to 1.23)^c^	142 434/8284	1.14 (1.12 to 1.18)^c^
***Women***					
**WC quintiles**					
Q1	70.0 (40.0-73.0)	46 406/2880	Ref.	46 406/3561	Ref.
Q2	76.0 (73.1-78.0)	38 788/2577	1.04 (0.99 to 1.10)	38 788/3158	1.04 (0.99 to 1.09)
Q3	81.0 (78.1-83.0)	33 802/2447	1.16 (1.06 to 1.18)	33 802/2979	1.10 (1.05 to 1.16)
Q4	87.0 (83.1-91.0)	39 450/2967	1.16 (1.10 to 1.22)	39 450/3640	1.15 (1.10 to 1.21)
Q5	98.0 (91.1-160.0)	38 310/2832	1.29 (1.22 to 1.36)	38 310/3456	1.27 (1.21 to 1.34)
***P* for trend/χ^2^**			<.001/306.77		<.001/345.67
**WC per SD increase** ^b^		196 756/13 703	1.13 (1.11 to 1.16)^c^	196 756/16 794	1.12 (1.10 to 1.15)^c^
**BMI quintiles**					
Q1	20.3 (15.0-21.5)	39 392/2063	Ref.	39 392/2567	Ref.
Q2	22.4 (21.6-23.2)	39 365/2541	1.03 (0.96 to 1.10)	39 365/3136	1.10 (1.05 to 1.16)
Q3	24.1 (23.3-25.1)	39 487/2884	1.11 (1.03 to 1.18)	39 487/3524	1.16 (1.10 to 1.22)
Q4	26.3 (25.2-27.9)	39 180/3084	1.24 (1.16 to 1.33)	39 180/3761	1.21 (1.15 to 1.27)
Q5	30.5 (28.0-60.0)	39 332/3131	1.34 (1.25 to 1.44)	39 332/3806	1.33 (1.26 to 1.40)
***P* for trend/χ^2^**			<.001/265.86		<.001/318.64
**BMI per SD increase** ^b^		196 756/13 703	1.13 (1.11 to 1.15)^c^	196 756/16 794	1.12 (1.09 to 1.14)^c^

Abbreviations: BMI = body mass index; CI = confidence interval; HR = hazard ratio; SD = standard deviation; WC = waist circumference.

a Hazard ratios were calculated by use of Cox regression using age as time scale, stratified by calendar year of birth (<1940, 1940-1949, 1950-1959, 1960-1969, ≥1970), and adjusted for baseline age (continuous), smoking status, cohort, marital status, education level, birth country, income level, and main source of income. We additionally adjusted for mode of waist circumference assessment and height (continuous) for the analysis of waist circumference, and mode of weight and height assessment for the analysis of body mass index. Waist circumference was standardized within sex strata.

b HRs per 1-SD increase were corrected for regression dilution ratios of body mass index or waist circumference (men, BMI: 0.92, WC: 0.78; women, BMI: 0.95, WC: 0.83).

c For all established obesity-related cancers, *P* values for heterogeneity between the HRs of WC and BMI were 0.014 in men and 0.357 in women. For all established and potential obesity-related cancers combined, the *P* values were 0.035 in men and 0.519 in women.

### WC as cancer risk factor beyond BMI (aim 2)

**Figure 3.**
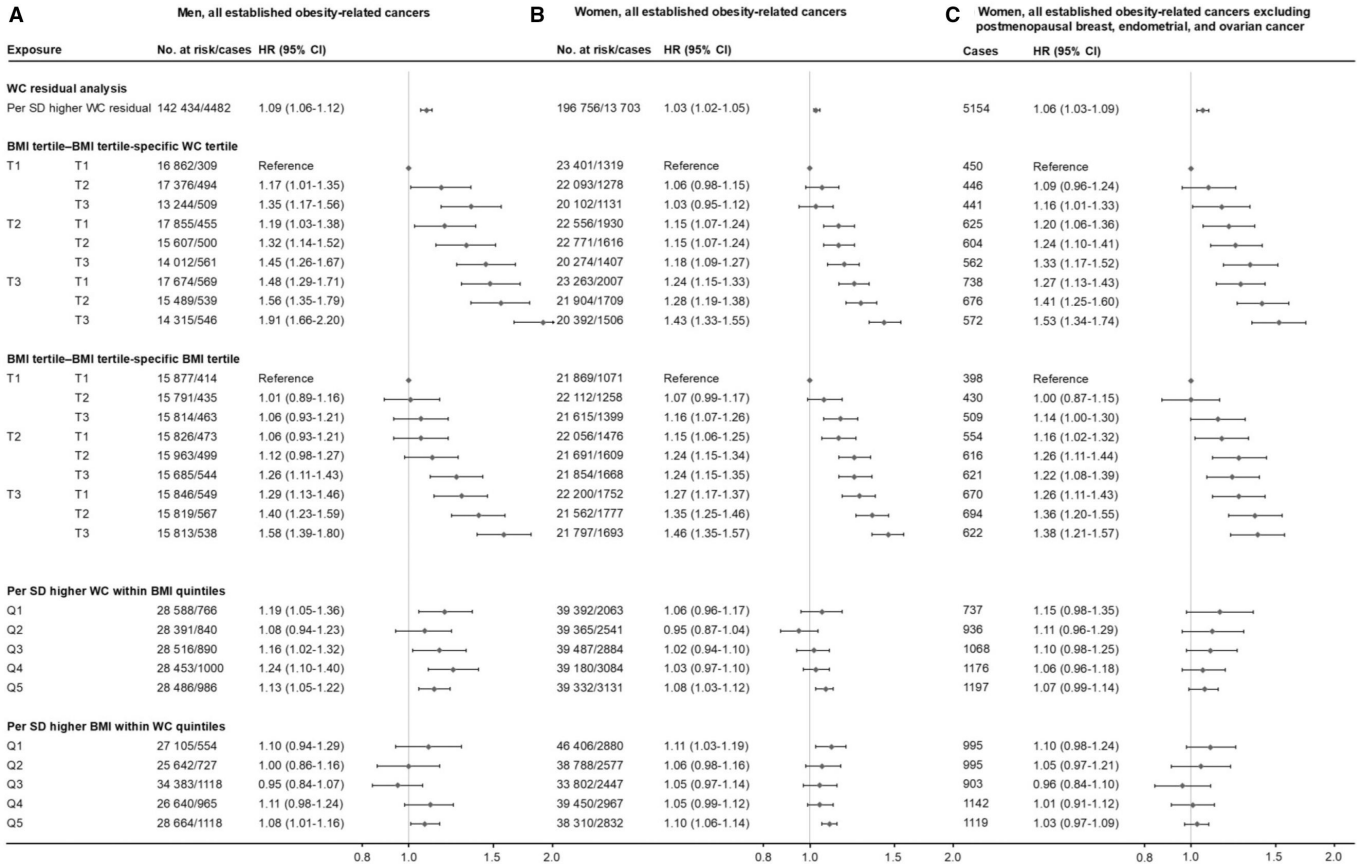
Hazard ratios (95% confidence interval) of all established obesity-related cancers combined associated with body mass index and waist circumference within body mass index and waist circumference tertiles and quintiles in men (A), women (B), and women excluding postmenopausal breast, endometrial, and ovarian cancer (C) separately. Waist circumference was standardized within sex strata. Hazard ratios were calculated by use of Cox regression using age as time scale, stratified by calendar year of birth (<1940, 1940-1949, 1950-1959, 1960-1969, ≥1970), and adjusted for baseline age (continuous), smoking status, cohort, marital status, education level, birth country, income level, and main source of income. We additionally adjusted for mode of waist circumference assessment and height (continuous) for the analysis of waist circumference, and mode of weight and height assessment for the analysis of body mass index. Abbreviations: BMI = body mass index; CI = confidence interval; HR = hazard ratio; SD = standard deviation; WC = waist circumference.

Residual analyses showed that WC residuals were associated with the risk of obesity-related cancer in men (HR per 1-SD = 1.09, 95% CI = 1.06 to 1.12) in multivariable, BMI-adjusted models. This association was weaker in women (HR = 1.03, 95% CI = 1.02 to 1.05) (Figure 3). After excluding postmenopausal breast and reproductive cancers, the results in women became slightly more similar to those in men (HR = 1.06, 95% CI = 1.03 to 1.09). Cancer site-specific analyses generally aligned with these findings (Table S3). Waist circumference residuals were not associated with postmenopausal breast cancer or endometrial cancer, the latter deviating from the general trend that cancers strongly associated with BMI also tended to show a stronger association with WC residuals (eg, cancers of the esophagus [adenocarcinoma], liver/intrahepatic bile ducts, renal cell, and pituitary gland).

There was a gradual increase in the risk of developing established obesity-related cancers across WC tertiles within BMI tertiles, for both men and women (Figure 3). Similarly, BMI tertiles within BMI tertiles also showed gradual increases in risk, which was expected due to the inherent gradient of BMI within BMI categories. Among men, the increase in risk was more pronounced for WC tertiles compared to BMI tertiles; all point estimates for the 8 HRs of WC tertile within BMI tertile combinations were higher than those for BMI tertile within BMI tertile combinations, although the 95% CIs overlapped. Specifically, the HR for the highest WC within the highest BMI group, compared to the lowest/lowest group, was 1.91 (95% CI = 1.66 to 2.20), whereas it was only 1.58 (95% CI = 1.39 to 1.80) for the highest BMI subgroup-tertile within the highest BMI group. In contrast, among women, HRs for WC tertiles within BMI tertiles were comparable to, or slightly smaller than, HRs for BMI subgroup-tertiles within BMI tertiles, but became slightly more similar to men’s results after excluding postmenopausal breast and reproductive cancers.

Analyses of continuous WC within BMI quintiles among men showed significant associations between WC and the risk of all established obesity-related cancers, except in the second-lowest BMI quintile. In contrast, continuous BMI within WC quintiles was not significantly associated with cancer risk, except in the highest WC quintile (Figure 3). Among women, both continuous WC within BMI quintiles and continuous BMI within WC quintiles exhibited weak and similarly sized associations with cancer risk.

Similar patterns were observed in the analysis of all potential and established obesity-related cancers combined (Figure S3).

## Discussion

In this prospective Swedish cohort of nearly 340 000 individuals, we found that WC was a stronger risk factor than BMI for obesity-related cancers in men. Additionally, in men, WC provided extra risk information beyond that of BMI alone, as confirmed by three distinct analytical approaches. In contrast, for women, the associations of WC and BMI with cancer risk were similar in magnitude, and WC provided little additional risk information beyond BMI.

Our findings are largely in line with the existing literature.^5^^,^^12-14^^,^^22^ Similar to a study conducted in the Alberta’s Tomorrow Project cohort,^5^ we found that WC was a stronger risk factor for cancer than BMI in men. However, most other studies have not shown this superiority, although the point estimates for WC were often slightly higher than those for BMI. For example, in a meta-analysis of 7 prospective cohorts investigating obesity-related cancers, the HRs per 1-SD increase were 1.11 for BMI and 1.13 for WC.^12^

There are several plausible explanations for this discrepancy: first, the joint analysis of men and women in other studies, and second, the higher intrapersonal variability of WC compared to BMI due to greater random measurement error, especially when self-reported. Previous studies have not accounted for this variability, potentially diminishing the differences between the observed effects of WC compared to BMI. In our study, the availability of repeated measurements allowed us to account for these dilution effects. Specifically, we observed RDR-corrected HRs of 1.19 (per 1-SD increase) for BMI and 1.25 for WC on established obesity-related cancers in men. Without RDR-correction, these values would have been 1.17 and 1.19, respectively, thus closer together.

Body mass index is a measure of body size, but does not provide information on fat distribution, whereas WC is a proxy more closely related to abdominal adiposity.^6^^,^^7^ This distinction is crucial because visceral fat, which accumulates around the abdominal organs, is more metabolically active and has been implicated in adverse health outcomes, including insulin resistance, inflammation, and dyslipidemia.^9-11^ Consequently, individuals with similar BMIs may have distinct cancer risks due to differences in fat distribution. Since visceral adiposity is a key determinant of insulin resistance, WC is likely a stronger risk factor than BMI. Our study partly confirms this, particularly for gastrointestinal cancers in men, although the overall epidemiological evidence does not fully support this hypothesis.^5^^,^^12-14^^,^^22^ One reason might be that WC captures not only visceral fat but also subcutaneous fat, which is much less relevant for the development of insulin resistance.^6^ For example, correlations of WC with visceral fat as determined via MRI measurements were only between 0.4 and 0.6 in a study by Grundy et al.^6^ Therefore, a more precise measure of visceral fat deposits is likely to yield stronger associations with cancer risk than BMI and WC. One promising approach could be to incorporate hip circumference, which is considered a more specific marker of subcutaneous and peripheral fat, into models evaluating the association of WC with cancer risk. In studies examining all-cause and cardiovascular disease mortality, adjusting for hip circumference substantially strengthened the association between WC and these outcomes.^23^^,^^24^ However, this approach has been less explored in relation to cancer risk,^12^ and future research is warranted to investigate the combined role of WC and hip circumference on cancer development.

An important aspect is the observed sex difference; WC appears to be a stronger risk factor than BMI for obesity-related cancers in men, but not in women. A plausible explanation is that men are more likely to store fat viscerally, while women generally accumulate more subcutaneous and peripheral fat.^25^ Consequently, WC is a more accurate measure of visceral fat in men than in women.^6^ This may make WC a stronger risk factor of cancer in men, and explain why WC adds risk information beyond that conveyed by BMI in men, but not women. Including hip circumference into risk models may provide further insights into this sex difference and enhance the association between WC and cancer, particularly for women. Additionally, research has indicated that adiposity, especially central adiposity, leads to higher concentrations of circulating insulin in men than in women. This may also partly explain why WC is more strongly associated with cancer risk in men.^26^^,^^27^ The divergence in how WC and BMI relate to cancer risk between men and women underscores the complexity of the impact of adiposity on cancer development. It suggests that considering biological and physiological differences between the sexes might be helpful when assessing cancer risk. Further research is needed to explore these sex differences.

Our findings suggest that WC may provide additional risk information for gastrointestinal cancers, such as colon cancer in men, but not for colon cancer and postmenopausal breast cancer in women, the 2 most common obesity-related cancers in women. This is consistent with previous reports comparing BMI and WC,^28-30^ and the established weaker link between obesity and colon cancer risk in women compared to men.^31^^,^^32^ It also aligns with our mechanistic understanding of how adiposity influences cancer risk. For gastrointestinal cancers, metabolic pathways and insulin resistance are likely relevant mediators. In contrast, for gynecological cancers, the pathway involving sex hormone alterations might be more relevant.^10^^,^^33^^,^^34^

The strengths of our study include the large sample size, long follow-up, and detailed cancer categorization. However, there are also potential limitations. As with all observational studies, there is the potential for residual confounding. We lacked information on certain lifestyle factors (eg, diet, alcohol consumption, physical activity), female reproductive factors (eg, parity, breastfeeding history), and history of viral infections (eg, human papillomavirus, Epstein–Barr virus, hepatitis), which we could not control for. Smoking data were limited to basic categories and were incomplete for some cohorts. However, sensitivity analyses in never-smokers and smoking-related cancers aligned with the main findings, suggesting that meaningful distortion from residual confounding or differential effects between BMI and WC is unlikely. We also did not have data on ethnicity, and while the vast majority of participants are likely Caucasian, the potential inclusion of individuals from other ethnic backgrounds may introduce a minor source of variability. Additionally, nearly 40% of WC values were self-reported. Self-reports are prone to social desirability bias, and although we corrected for regression dilution bias, some bias likely remains. Still, subgroup analyses by mode of measurement revealed consistent findings, although HRs for WC were attenuated in the self-reported subgroup, highlighting the potential influence of measurement error, particularly for WC. Furthermore, while our primary models assumed linearity, this approach may not have fully captured nonlinear associations for rarer cancers. Finally, BMI and WC are surrogate measures of general and abdominal adiposity; more precise measures of adiposity, such as dual-energy X-ray absorptiometry or the inclusion of hip circumference, might reveal stronger associations.

In conclusion, our study provides evidence that WC is a stronger risk factor than BMI for obesity-related cancers in men, but not in women. Additionally, WC appears to provide additional risk information beyond that conveyed by BMI in men. The weaker contribution of WC beyond that of BMI in women may result from sex-specific interactions with adiposity measures or differences in how WC and BMI reflect body fat in men vs women. Future research incorporating more precise measures of adiposity, along with comprehensive data on potential confounding factors, could further elucidate the relationship between body fat distribution and cancer risk.

## Supplementary Material

djaf075_Supplementary_Data

## Data Availability

All data are located on Statistics Sweden’s Microdata Online Access server and may only be accessed from countries in the European Union or the European Economic Area. Data access covered by the ethical approval will be considered in agreement with the principal investigator of ODDS, T.S., and upon approval from register holders and steering committees of ODDS cohorts.
