# Supplementary material for: Comparing waist circumference with body mass index on obesity-related cancer risk: a pooled Swedish study
Source: J Natl Cancer Inst. 2025 Mar 28;117(10):1999–2009. doi: 10.1093/jnci/djaf075 (PMC12505136; doi:10.1093/jnci/djaf075)
Supplement: djaf075_Supplementary_Data [file djaf075_supplementary_data.pdf]

Supplementary Material

Comparing waist circumference with body mass index on obesity-related cancer risk: a pooled Swedish study

Ming Sun, Christel Häggström, Marisa da Silva, Innocent B. Mboya, Ylva Trolle Lagerros, Karl Michaëlsson, Sven Sandin, Jerzy Leppert, Sara Hägg, Sölve Elmståhl, Patrik K. E. Magnusson, Stefan Söderberg, Weiyao Yin, Abbas Chabok, Angela Wood, Tanja Stocks, Josef Fritz

Corresponding author: Josef Fritz

Email: josef.fritz@med.lu.se

Table of contents

Supplementary Table 1. The codes according to the International Classification of Diseases, the International Classification of Diseases for Oncology, and WHO/HS/CANC/24·1 (Swedish PAD codes) used to categorize all cancers which have been analyzed in the study 3

Supplementary Table 2. Hazard ratios (95% confidence interval) of all established obesity-related cancers and all established and potential obesity-related cancers combined according to body mass index and waist circumference level in the full population, in never-smokers only, and by mode of measurement (objectively measured vs. self-reported data) 4

Supplementary Table 3. Hazard ratios (95% confidence interval) of each established and potential obesity-related cancer per standard deviation higher waist circumference residual, in men and women combined and separately 6

Supplementary Figure 1. Flowchart of exclusions and selection of individuals and observations in the study 7

Supplementary Figure 2. Overall and sex and time-specific regression dilution ratios (95% confidence intervals) of body mass index and waist circumference 8

Supplementary Figure 3. Hazard ratios (95% confidence interval) of all established and potential obesity-related cancers combined associated with body mass index and waist circumference within body mass index and waist circumference tertiles and quintiles in men (A) and women (B) separately9

Supplementary Table 1. The codes according to the International Classification of Diseases, the International Classification of Diseases for Oncology, and WHO/HS/CANC/24·1 (Swedish PAD codes) used to categorize all cancers which have been analyzed in the study

Cancer category	ICD7 (since 1958)	ICD10 (since 1993)	ICD-O-2 (since 1993)	C24.1 (since 1958)
Oral cavity	140, 141.7-9,143-144	C00, C02-04, C05.0, C06		
Nasal and paranasal sinuses[1]	160	C30-31		
Head and neck, adenocarcinoma[1,2]	140,141.0, 141.7-9, 142-144, 145.0, 145.7-9, 146-148, 160-161	C00, C01.9, C02-04, C05-14, C30-C32	81403	
Head and neck, squamous-cell carcinoma[1,3]	140,141.0, 141.7-9, 142-144, 145.0, 145.7-9, 146-148, 160-161	C00, C01.9, C02-04, C05-14, C30-C32		146
Esophagus, adenocarcinoma	150	C15		096
Gastric, cardia	151.1	C16.0		
Gastric, gastrointestinal stromal[1]	151	C16		796
Small intestine	152	C17		
Colon	153	C18		
Rectum/anus	154	C19-21		
Liver/intrahepatic bile ducts	155.0, 156	C22		
Biliary tract	155.1-9	C23-24		
Gallbladder	155.1	C23		
Pancreas	157	C25.0-3, C25.8-9		
Malignant melanoma[2]	190	C43		
Nodular[3]			87213	
Breast (postmenopausal was analyzed)	170	C50		
Vulva[3]	176.0, 176.7-8	C51		
Cervix, adenocarcinoma[1,3]	171	C53		096
Endometrium		C54.1		
Ovary	175.0	C56		
Penis[1,2]	179.0	C60		
Renal cell	180.0	C64.0		
Meningioma	192.1, 193	C47, C70-72, C75.2, C75.3		461, 463, 466
Pancreatic islets[1]	195.5	C25.4		
Thyroid	194	C73		
Adrenal glands[1]	195.0	C74		
Parathyroid gland	195.1	C75.0		
Pituitary gland	195.3	C75.1		
Connective tissue	197.0-3, 197.7-9	C49		
Lymphoid neoplasms	200-202, 204	C81-85, C88, C91, C96		
Multiple myeloma	203	C90		
Myeloid neoplasms	205-207.3, 209	C92-95, D46, D47.1		

Abbreviations: ICD, International Classification of Diseases.

Italic and bold letters indicate IARC-established obesity-related cancers. In the Swedish Cancer Register, all codes are converted into the ICD-7 edition for recording. However, the categorization is more detailed for later ICD editions than for ICD-7. Therefore, we used the oldest ICD edition available through follow-up for cancer for which we judged the level of detail of the ICD edition to be sufficient.

[1]Not analyzed separately due to a small number of cases (<100), but included in the analyses of established and potential obesity-related cancers combined.

[2]Considered potentially obesity-related only in men and therefore included exclusively in analyses of men.

[3]Considered potentially obesity-related only in women and therefore included exclusively in analyses of women.

Supplementary Table 2. Hazard ratios (95% confidence interval) of all established obesity-related cancers and all established and potential obesity-related cancers combined according to body mass index and waist circumference level in the full population, in never-smokers only, and by mode of measurement (objectively measured vs. self-reported data)

	All established obesity-related cancers		All established and potential obesity-related cancers	
	No. at risk/cases	HR (95% CI)[1]	No. at risk/cases	HR (95% CI)[1]
Full population, all cancers (as reported in Table 2)				
Men				
WC per SD increase	142 434/4482	1.25 (1.21-1.30)	142 434/8284	1.18 (1.16-1.22)
BMI per SD increase	142 434/4482	1.19 (1.15-1.23)	142 434/8284	1.14 (1.12-1.18)
Women				
WC per SD increase	196 756/13 703	1.13 (1.11-1.16)	196 756/16 794	1.12 (1.10-1.15)
BMI per SD increase	196 756/13 703	1.13 (1.11-1.15)	196 756/16 794	1.12 (1.09-1.14)
Never-smokers, all cancers				
Men				
WC per SD increase	61 578/1430	1.21 (1.12-1.29)	61 578/2907	1.18 (1.12-1.24)
BMI per SD increase	61 578/1430	1.18 (1.11-1.25)	61 578/2907	1.14 (1.10-1.20)
Women				
WC per SD increase	94 224/6329	1.15 (1.11-1.18)	94 224/7830	1.13 (1.11-1.17)
BMI per SD increase	94 224/6329	1.13 (1.09-1.16)	94 224/7830	1.12 (1.09-1.15)
Full population, smoking-related cancers[2]				
Men				
WC per SD increase	142 434/1043	1.37 (1.26-1.48)	142 434/1263	1.29 (1.20-1.39)
BMI per SD increase	142 434/1043	1.24 (1.16-1.33)	142 434/1263	1.20 (1.13-1.27)
Women				
WC per SD increase	196 756/895	1.16 (1.07-1.27)	196 756/1267	1.16 (1.07-1.23)
BMI per SD increase	196 756/895	1.09 (1.02-1.18)	196 756/1267	1.09 (1.02-1.16)
Never-smokers, smoking-related cancers[2]				
Men				
WC per SD increase	61 578/276	1.39 (1.18-1.62)	61 578/330	1.33 (1.14-1.55)
BMI per SD increase	61 578/276	1.23 (1.08-1.41)	61 578/330	1.22 (1.04-1.37)
Women				
WC per SD increase	94 224/366	1.17 (1.02-1.35)	94 224/512	1.20 (1.10-1.35)
BMI per SD increase	94 224/366	1.11 (0.99-1.24)	94 224/512	1.11 (1.01-1.22)
Individuals with objectively measured WC and BMI values, all cancers				
Men				
WC per SD increase	98 303/2355	1.28 (1.21-1.34)	98 303/4413	1.20 (1.14-1.24)
BMI per SD increase	98 303/2355	1.19 (1.13-1.23)	98 303/4413	1.13 (1.10-1.18)
Women				
WC per SD increase	107 217/5997	1.16 (1.12-1.20)	107 217/7306	1.15 (1.12-1.18)
BMI per SD increase	107 217/5997	1.13 (1.09-1.16)	107 217/7306	1.12 (1.09-1.15)
Individuals with self-reported WC and BMI values, all cancers				
Men				
WC per SD increase	44 131/2127	1.22 (1.16-1.30)	44 131/3871	1.18 (1.13-1.24)
BMI per SD increase	44 131/2127	1.19 (1.12-1.24)	44 131/3871	1.14 (1.11-1.20)
Women				
WC per SD increase	89 539/7706	1.11 (1.07-1.13)	89 539/9488	1.10 (1.07-1.13)
BMI per SD increase	89 539/7706	1.11 (1.08-1.14)	89 539/9488	1.11 (1.07-1.13)

BMI, body mass index; WC, waist circumference; HR, hazard ratio; CI, confidence interval; SD, standard deviation.

[1]Hazard ratios are given per 1-standard deviation increase and derived from Cox regression models using age as time scale, stratified by calendar year of birth (<1940, 1940-1949, 1950-1959, 1960-1969, ≥1970), and adjusted for baseline age (continuous), smoking status, cohort, marital status, education level, birth country, income level, and main source of income. We additionally adjusted for mode of waist circumference assessment and height (continuous) for the analysis of waist circumference, and mode of weight and height assessment for the analysis of body mass index. Waist circumference was standardized within sex strata. HRs per 1-SD increase

were corrected for regression dilution ratios of body mass index or waist circumference (men, BMI: 0.92, WC: 0.78; women, BMI: 0.95, WC: 0.83).

[2]Smoking-related cancers include cancers of the oral cavity, nasal and paranasal sinuses, head and neck (adenocarcinoma and squamous cell carcinoma), oesophagus (adenocarcinoma), stomach-cardia, liver/intrahepatic bile ducts, and pancreas.

Supplementary Table 3. Hazard ratios (95% confidence interval) of each established and potential obesity-related cancer per standard deviation higher waist circumference residual, in men and women combined and separately

	All		Men		Women	
**Cancer type**	**No. at risk/cases**	**HR (95% CI)[1]**	**No. at risk/cases**	**HR (95% CI)[1]**	**No. at risk/cases**	**HR (95% CI)[1]**
Oral cavity	339 190/421	1.01 (0.93-1.11)	142 434/185	0.94 (0.82-1.07)	196 756/236	1.08 (0.96-1.23)
Esophagus (adenocarcinoma)	339 190/189	**1.24 (1.09-1.41)**	142 434/151	**1.19 (1.03-1.38)**	196 756/38	NA[2]
Gastric (cardia)	339 190/223	1.10 (0.97-1.24)	142 434/158	1.08 (0.93-1.26)	196 756/65	NA[2]
Small intestine	339 190/239	1.11 (0.99-1.25)	142 434/125	1.07 (0.91-1.25)	196 756/114	1.16 (0.98-1.38)
Colon	339 190/3717	NA[3]	142 434/1642	**1.08 (1.03-1.13)**	196 756/2075	**1.04 (1.00-1.08)**
Rectum	339 190/1957	**1.06 (1.02-1.11)**	142 434/1058	1.05 (0.99-1.11)	196 756/899	**1.09 (1.02-1.16)**
Liver/intrahepatic bile ducts	339 190/458	**1.12 (1.03-1.22)**	142 434/255	**1.15 (1.02-1.28)**	196 756/203	1.09 (0.96-1.25)
Biliary tract	339 190/413	1.05 (0.96-1.15)	142 434/141	1.11 (0.95-1.30)	196 756/272	1.02 (0.91-1.13)
Gallbladder	339 190/209	0.94 (0.83-1.06)	142 434/41	NA[2]	196 756/168	0.94 (0.82-1.08)
Pancreas	339 190/1069	**1.07 (1.01-1.13)**	142 434/479	**1.09 (1.00-1.19)**	196 756/590	1.05 (0.97-1.13)
Melanoma	339 190/2737	NA[4]	142 434/1250	1.03 (0.98-1.09)	196 756/1487	NA[4]
Melanoma (nodular)	338 704/327	1.05 (0.95-1.17)	142 233/199	NA[4]	196 471/128	**1.15 (1.00-1.32)**
Breast (postmenopausal)	-	-	-	-	156 760/6851	1.01 (0.94-1.07)
Vulva	-	-	-	-	196 756/172	**1.11 (1.01-1.22)**
Endometrium	-	-	-	-	196 471/889	1.03 (0.94-1.13)
Ovary	-	-	-	-	196 756/858	**1.12 (1.00-1.25)**
Renal cell	339 190/899	**1.15 (1.08-1.22)**	142 434/516	**1.17 (1.08-1.27)**	196 756/383	**1.11 (1.00-1.23)**
Meningioma	339 190/532	1.02 (0.93-1.11)	142 434/112	0.96 (0.80-1.15)	196 756/420	1.10 (0.94-1.28)
Thyroid	339 190/373	**1.12 (1.02-1.24)**	142 434/87	NA[2]	196 756/286	1.06 (0.90-1.24)
Parathyroid gland	339 190/377	**1.13 (1.02-1.24)**	142 434/57	NA[2]	196 756/320	0.99 (0.94-1.05)
Pituitary gland	339 190/295	**1.15 (1.03-1.28)**	142 434/135	**1.20 (1.03-1.41)**	196 756/160	1.05 (0.95-1.17)
Connective tissue	339 190/270	**1.13 (1.01-1.27)**	142 434/135	**1.22 (1.04-1.43)**	196 756/135	0.97 (0.88-1.07)
Lymphoid neoplasms	339 190/2362	1.02 (0.99-1.07)	142 434/1311	**1.05 (1.00-1.11)**	196 756/1051	1.08 (0.90-1.28)
Multiple myeloma	339 190/718	1.03 (0.96-1.10)	142 434/375	1.01 (0.91-1.11)	196 756/343	1.04 (0.99-1.11)
Myeloid neoplasms	339 190/712	1.03 (0.96-1.10)	142 434/342	1.10 (0.99-1.22)	196 756/370	1.01 (0.99-1.04)

BMI, body mass index; CI, confidence interval; NA, not available; HR, hazard ratio.

[1] Waist circumference was regressed on body mass index in sex-specific, multivariable-adjusted linear regression models. The residuals from these models were then included in a Cox model (using age as time scale) also adjusted for BMI, mode of waist circumference assessment, baseline age (continuous), smoking status, cohort, marital status, education level, birth country, income level, and main source of income, and stratified by calendar year of birth (<1940, 1940-1949, 1950-1959, 1960-1969, ≥1970) and sex (in analyses of men and women combined). The HRs reported here are the per standard deviation higher waist circumference residual HRs from these Cox models.

[2] The number of cancer cases was considered too low for analysis (< 100 cases).

[3] P sex-interaction<0.05, calculated by adding a product term of sex and BMI in categories or per 5 kg/m^2 higher BMI in the Cox model using the Wald test.

[4] The cancer is not obesity-related.

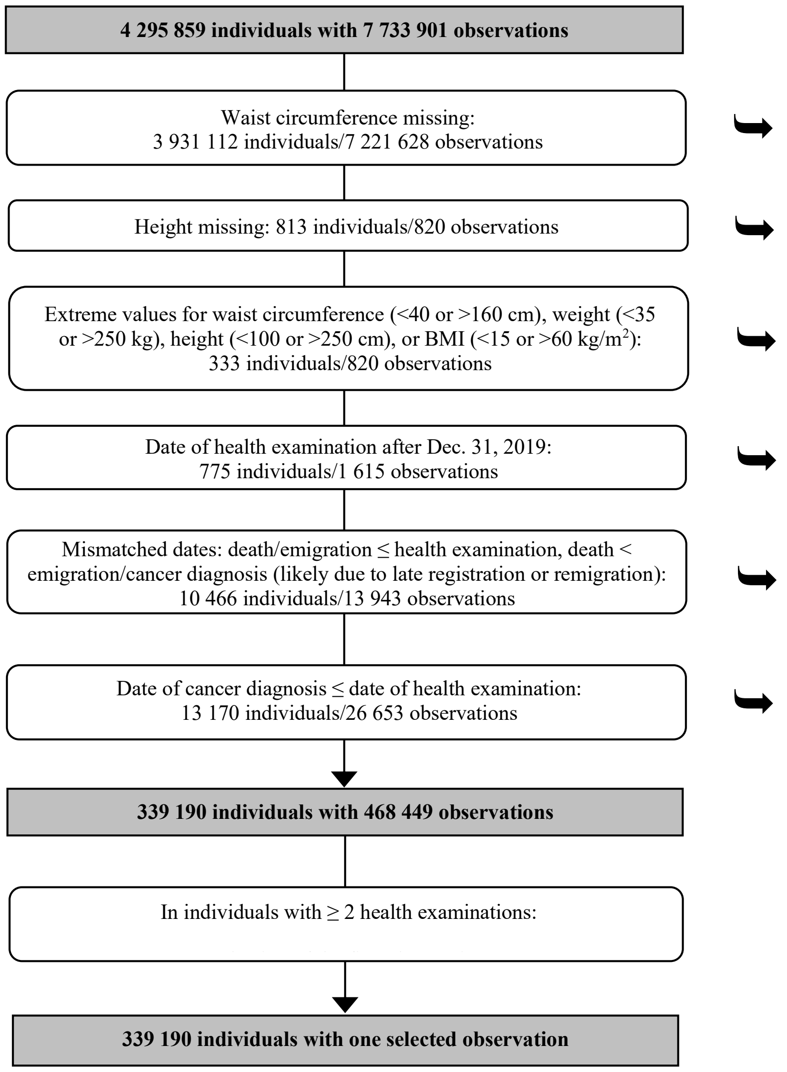

Supplementary Figure 1. Flowchart of exclusions and selection of individuals and observations in the study

➥ denotes exclusions

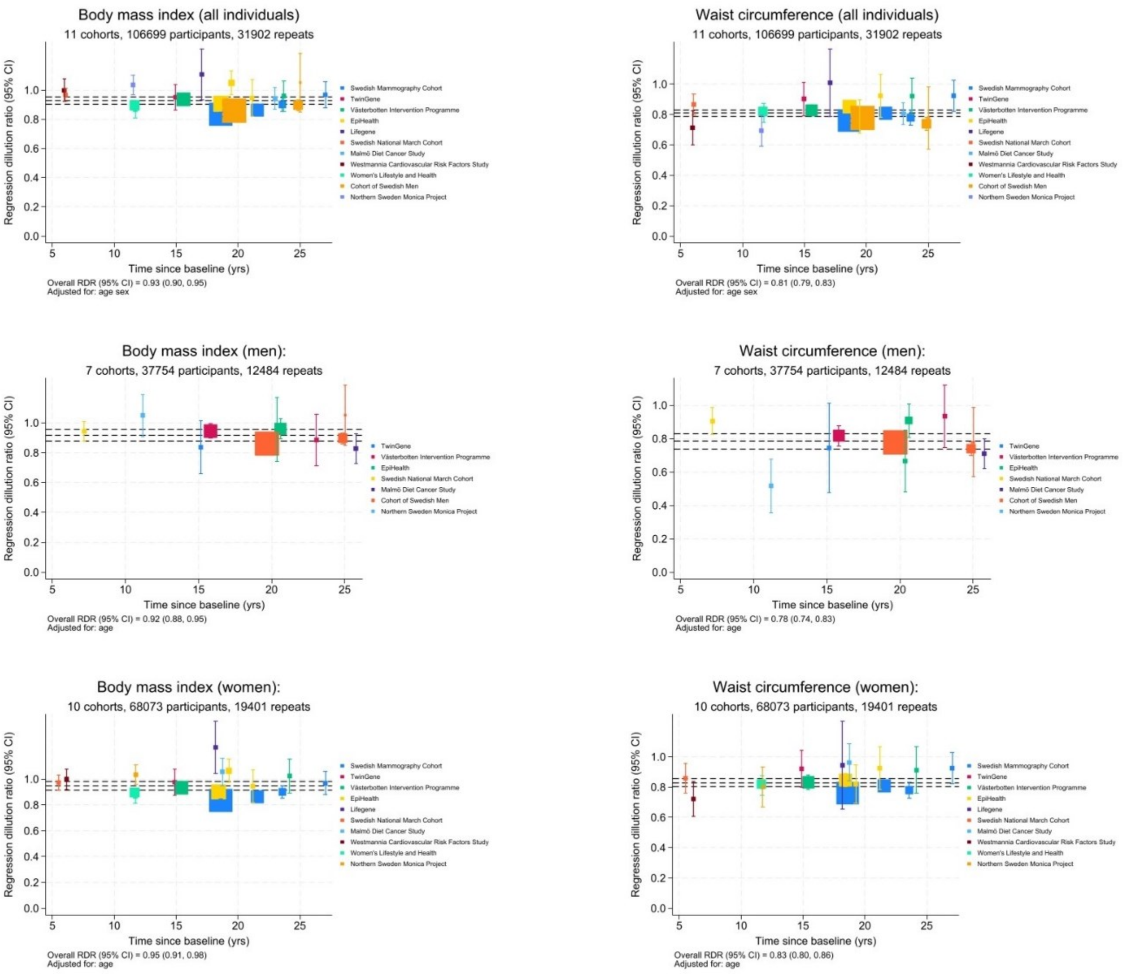

Supplementary Figure 2. Overall and sex and time-specific regression dilution ratios (95% confidence intervals) of body mass index and waist circumference

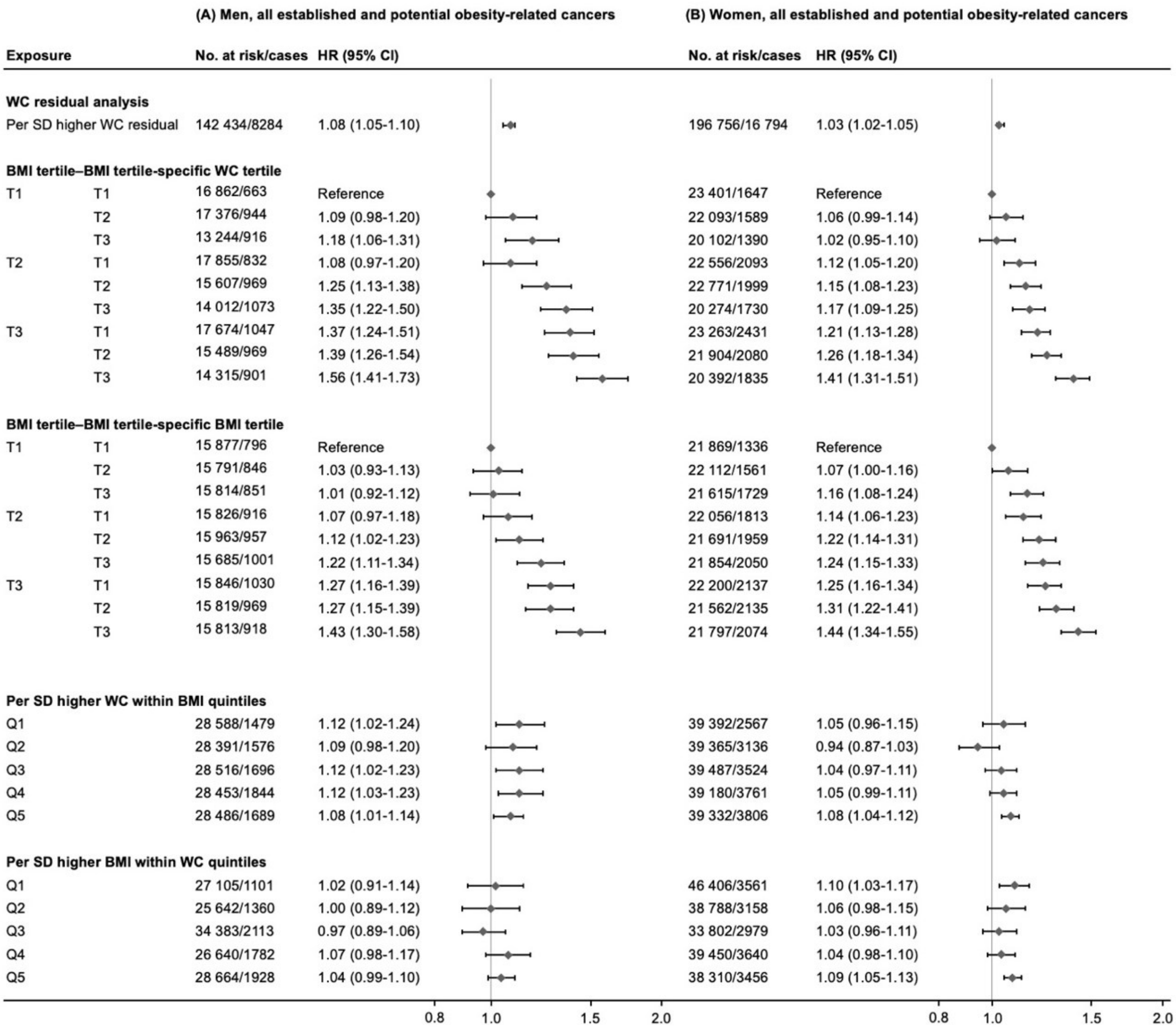

Exposure		(A) Men, all established and potential obesity-related cancers: No. at risk/cases	(A) HR (95% CI)	(B) Women, all established and potential obesity-related cancers: No. at risk/cases	(B) HR (95% CI)
**WC residual analysis**					
Per SD higher WC residual		142 434/8284	1.08 (1.05-1.10)	196 756/16 794	1.03 (1.02-1.05)
**BMI tertile–BMI tertile-specific WC tertile**					
T1	T1	16 862/663	Reference	23 401/1647	Reference
	T2	17 376/944	1.09 (0.98-1.20)	22 093/1589	1.06 (0.99-1.14)
	T3	13 244/916	1.18 (1.06-1.31)	20 102/1390	1.02 (0.95-1.10)
T2	T1	17 855/832	1.08 (0.97-1.20)	22 556/2093	1.12 (1.05-1.20)
	T2	15 607/969	1.25 (1.13-1.38)	22 771/1999	1.15 (1.08-1.23)
	T3	14 012/1073	1.35 (1.22-1.50)	20 274/1730	1.17 (1.09-1.25)
T3	T1	17 674/1047	1.37 (1.24-1.51)	23 263/2431	1.21 (1.13-1.28)
	T2	15 489/969	1.39 (1.26-1.54)	21 904/2080	1.26 (1.18-1.34)
	T3	14 315/901	1.56 (1.41-1.73)	20 392/1835	1.41 (1.31-1.51)
**BMI tertile–BMI tertile-specific BMI tertile**					
T1	T1	15 877/796	Reference	21 869/1336	Reference
	T2	15 791/846	1.03 (0.93-1.13)	22 112/1561	1.07 (1.00-1.16)
	T3	15 814/851	1.01 (0.92-1.12)	21 615/1729	1.16 (1.08-1.24)
T2	T1	15 826/916	1.07 (0.97-1.18)	22 056/1813	1.14 (1.06-1.23)
	T2	15 963/957	1.12 (1.02-1.23)	21 691/1959	1.22 (1.14-1.31)
	T3	15 685/1001	1.22 (1.11-1.34)	21 854/2050	1.24 (1.15-1.33)
T3	T1	15 846/1030	1.27 (1.16-1.39)	22 200/2137	1.25 (1.16-1.34)
	T2	15 819/969	1.27 (1.15-1.39)	21 562/2135	1.31 (1.22-1.41)
	T3	15 813/918	1.43 (1.30-1.58)	21 797/2074	1.44 (1.34-1.55)
**Per SD higher WC within BMI quintiles**					
Q1		28 588/1479	1.12 (1.02-1.24)	39 392/2567	1.05 (0.96-1.15)
Q2		28 391/1576	1.09 (0.98-1.20)	39 365/3136	0.94 (0.87-1.03)
Q3		28 516/1696	1.12 (1.02-1.23)	39 487/3524	1.04 (0.97-1.11)
Q4		28 453/1844	1.12 (1.03-1.23)	39 180/3761	1.05 (0.99-1.11)
Q5		28 486/1689	1.08 (1.01-1.14)	39 332/3806	1.08 (1.04-1.12)
**Per SD higher BMI within WC quintiles**					
Q1		27 105/1101	1.02 (0.91-1.14)	46 406/3561	1.10 (1.03-1.17)
Q2		25 642/1360	1.00 (0.89-1.12)	38 788/3158	1.06 (0.98-1.15)
Q3		34 383/2113	0.97 (0.89-1.06)	33 802/2979	1.03 (0.96-1.11)
Q4		26 640/1782	1.07 (0.98-1.17)	39 450/3640	1.04 (0.98-1.10)
Q5		28 664/1928	1.04 (0.99-1.10)	38 310/3456	1.09 (1.05-1.13)

Supplementary Figure 3. Hazard ratios (95% confidence interval) of all established and potential obesity-related cancers combined associated with body mass index and waist circumference within body mass index and waist circumference tertiles and quintiles in men (A) and women (B) separately

Waist circumference was standardised within sex strata. Hazard ratios were calculated by use of Cox regression using age as time scale, stratified by calendar year of birth (<1940, 1940-1949, 1950-1959, 1960-1969, ≥1970), and adjusted for baseline age (continuous), smoking status, cohort, marital status, education level, birth country, income level, and main source of income. We additionally adjusted for mode of waist circumference assessment and height (continuous) for the analysis of waist circumference, and mode of weight and height assessment for the analysis of body mass index. HR, hazard ratio; CI, confidence interval; SD, standard deviation; WC, waist circumference; BMI, body mass index.
